# Violence against children in Latin America and the Caribbean: What do available data reveal about prevalence and perpetrators?

**DOI:** 10.26633/RPSP.2019.66

**Published:** 2019-10-15

**Authors:** Karen Devries, Katherine G Merrill, Louise Knight, Sarah Bott, Alessandra Guedes, Betzabe Butron-Riveros, Constanza Hege, Max Petzold, Amber Peterman, Claudia Cappa, Lauren Maxwell, Abigail Williams, Sunita Kishor, Naeemah Abrahams

**Affiliations:** 1 London School of Hygiene & Tropical Medicine London School of Hygiene & Tropical Medicine Department of Global Health and Development London United Kingdom Department of Global Health and Development, London School of Hygiene & Tropical Medicine, London, United Kingdom.; 2 Johns Hopkins Bloomberg School of Public Health Johns Hopkins Bloomberg School of Public Health Department of International Health BaltimoreMaryland United States of America Department of International Health, Johns Hopkins Bloomberg School of Public Health, Baltimore, Maryland, United States of America.; 3 Pan American Health Organization (PAHO) Pan American Health Organization (PAHO) WashingtonDC United States Pan American Health Organization (PAHO), Washington, DC, United States.; 4 Institute of Medicine, University of Gothenburg Institute of Medicine, University of Gothenburg School of Public Health and Community Medicine Gothenburg Sweden School of Public Health and Community Medicine, Institute of Medicine, University of Gothenburg, Gothenburg, Sweden.; 5 United Nations Children’s Fund (UNICEF), Office of Research–Innocenti United Nations Children’s Fund (UNICEF), Office of Research–Innocenti Social and Economic Policy Unit Florence Italy Social and Economic Policy Unit, United Nations Children’s Fund (UNICEF), Office of Research–Innocenti, Florence, Italy.; 6 Research and Policy, New York, UNICEF Research and Policy, New York, UNICEF Data and Analytics Section, Division of Data New YorkD.C. United States Data and Analytics Section, Division of Data, Research and Policy, New York, UNICEF, New York, United States.; 7 Hubert Department of Global Health, Emory University Hubert Department of Global Health, Emory University Atlanta Georgia Hubert Department of Global Health, Emory University, Atlanta, Georgia, United States.; 8 King’s College London King’s College London London United Kingdom King’s College London, London, United Kingdom.; 9 The DHS Program, ICF International, Fairfax The DHS Program, ICF International, Fairfax Virginia United States The DHS Program, ICF International, Fairfax, Virginia, United States.; 10 South African Medical Research Council South African Medical Research Council Gender and Health Research Unit Cape Town South Africa Gender and Health Research Unit, South African Medical Research Council, Cape Town, South Africa.

**Keywords:** Child abuse, physical abuse, violence, child health, adolescent health, Latin America, Caribbean Region, Maltrato a los niños, abuso físico, violencia, salud del niño, salud del adolescente, América Latina, Región del Caribe, Maus-tratos infantis, abuso físico, violência, saúde da criança, saúde do adolescente, América Latina, Região do Caribe

## Abstract

**Objective.:**

To describe the prevalence of recent physical, sexual, and emotional violence against children 0 – 19 years of age in Latin America and the Caribbean (LAC) by age, sex, and perpetrator.

**Methods.:**

A systematic review and analysis of published literature and large international datasets was conducted. Eligible sources from first record to December 2015 contained age-, sex-, and perpetrator-specific data from LAC. Random effects meta-regressions were performed, adjusting for relevant quality covariates and differences in violence definitions.

**Results.:**

Seventy-two surveys (2 publications and 70 datasets) met inclusion criteria, representing 1 449 estimates from 34 countries. Prevalence of physical and emotional violence by caregivers ranged from 30% – 60%, and decreased with increasing age. Prevalence of physical violence by students (17% – 61%) declined with age, while emotional violence remained constant (60% – 92%). Prevalence of physical intimate partner violence (IPV) ranged from 13% – 18% for girls aged 15 – 19 years. Few or no eligible past-year estimates were available for any violence against children less than 9 years and boys 16 – 19 years of age; sexual violence against boys (any age) and girls (under 15 years); IPV except for girls aged 15 – 19 years; and violence by authority figures (e.g., teachers) or via gangs/organized crime.

**Conclusion.:**

Past-year physical and emotional violence by caregivers and students is widespread in LAC across all ages in childhood, as is IPV against girls aged 15 – 19 years. Data collection must be expanded in LAC to monitor progress towards the sustainable development goals, develop effective prevention and response strategies, and shed light on violence relating to organized crime/gangs.

## RESUMEN

**Objetivo.:**

Describir la prevalencia de la violencia física, sexual y emocional infligida recientemente en niños y niñas de 0 a 19 años en América Latina y el Caribe (ALC) según la edad, el sexo y el agresor.

**Métodos.:**

Se llevó a cabo una revisión y un análisis sistemáticos de la bibliografía publicada en los grandes conjuntos de datos. Las fuentes desde los primeros registros hasta diciembre del 2015 que cumplían los criterios fueron las que contenían datos específicos de América Latina y el Caribe en función de la edad, el sexo y el agresor. Se realizaron metarregresiones de los efectos aleatorios, con ajustes pertinentes para las covariables de calidad y las diferencias en las definiciones de violencia.

**Resultados.:**

Setenta y dos encuestas (2 publicaciones y 70 conjuntos de datos) cumplieron los criterios de inclusión, que representaban 1.449 estimaciones de 34 países. La prevalencia de la violencia física y emocional infligida por cuidadores fue del 30% al 60% y disminuyó al aumentar la edad. La prevalencia de la violencia física infligida por estudiantes (17% a 61%) disminuyó con la edad, mientras que la violencia emocional se mantuvo constante (60% a 92%). La prevalencia de la violencia física infligida por la pareja fue de 13% a 18% para las niñas en edades de 15 a 19 años. Fueron pocas o inexistentes las estimaciones que cumplieran los criterios realizadas el año pasado sobre: la violencia de todo tipo contra los niños y las niñas menores de 9 años y los niños entre los 16 y 19 años; la violencia sexual contra los niños de cualquier edad y las niñas menores de 15 años; la violencia infligida por la pareja, salvo en niñas de 15 a 19 años; y la violencia infligida por figuras de autoridad (por ejemplo profesores) o por pandillas o el crimen organizado.

**Conclusión.:**

La violencia emocional y física infligida por los cuidadores y estudiantes es generalizada en América Latina y el Caribe en todas las edades en la niñez, al igual que la violencia infligida por la pareja hacia niñas en edades entre los 15 y 19 años. Se debe ampliar la recopilación de datos en América Latina y el Caribe para dar seguimiento al progreso hacia los objetivos de desarrollo sostenible, elaborar estrategias eficaces de prevención y respuesta, y arrojar luz sobre la violencia en relación con el crimen organizado y las pandillas.

## RESUMO

**Objetivo.:**

Descrever a prevalência da violência física, sexual e emocional recente contra crianças entre 0 e 19 anos de idade na América Latina e no Caribe (ALC), discriminada por idade, sexo e autor da agressão.

**Métodos.:**

Foi realizada uma revisão sistemática e análise da literatura publicada e de grandes conjuntos de dados internacionais. As fontes qualificadas do primeiro registro de dezembro de 2015 continham dados provenientes da ALC, discriminados por idade, sexo e autor da agressão. Foi usada a técnica de metarregressão de efeitos aleatórios, com ajuste para covariáveis de interesse de qualidade e para diferenças nas definições de violência.

**Resultados.:**

Setenta e dois levantamentos (2 publicações e 70 conjuntos de dados) satisfizeram os critérios de inclusão, representando 1.449 estimativas provenientes de 34 países. A prevalência da violência física e emocional por cuidadores variou de 30% a 60%, sendo que este percentual diminuiu com o aumento da idade. A prevalência da violência física escolar (17% a 61%) diminuiu com a idade e a prevalência da violência infantil emocional ficou constante (60% a 92%). A prevalência da violência física por parceiro íntimo variou de 13% a 18% nas adolescentes entre 15 e 19 anos de idade. Foram encontradas poucas estimativas para o ano precedente, ou as estimativas existentes não satisfizeram os critérios, quanto à violência infantil em crianças menores de 9 anos e adolescentes do sexo masculino entre 16 e 19 anos de idade, à violência sexual contra meninos (em qualquer idade) e meninas (menores de 15 anos), à violência por parceiro íntimo, exceto em meninas entre 15 e 19 anos de idade e à violência infligida por figuras de autoridade (como professores) ou gangues/crime organizado.

**Conclusão.:**

A violência física e emocional no ano precedente praticada por cuidadores e na escola é generalizada na ALC e ocorre em todas as faixas etárias na infância, assim como a violência por parceiro íntimo contra as adolescentes entre 15 e 19 anos de idade. A coleta de dados deve ser ampliada na ALC para monitorar o progresso rumo ao alcance dos objetivos de desenvolvimento sustentável, criar estratégias efetivas de prevenção e resposta à violência e identificar a violência relativa ao crime organizado/quadrilhas.

Violence in childhood is a global health and human rights issue. Latin America and the Caribbean (LAC) are recognized as among the most violent geographic areas globally, particularly for young people (1, 2). An estimated 58% of children 0 – 17 years of age in LAC (more than 99 million) experience physical, sexual, or emotional abuse each year (3). Health consequences include physical injury, mental health problems, and increased risk of substance use, among others (4 – 6). Violence drains the health, social, and judicial sector budgets, with expenditures for treating survivors and prosecuting perpetrators (7). Furthermore, early exposure to violence has been linked to multiple forms of violence perpetration and victimization in adulthood (8).

Preventing and responding to violence against children is a global priority, as evidenced by the 2030 Sustainable Development Goals (SDGs) and the priorities of the Pan American Health Organization (PAHO) and LAC governments (9). All LAC countries are signatories to the United Nations Convention on the Rights of the Child (10) and have supported the Organization of American States’ resolutions on violence and human rights (11, 12). Many countries have developed national laws and policies to address violence in childhood; for example, at least 10 LAC countries prohibit corporal punishment in all settings (13).

To effectively prevent and respond to violence in childhood, an understanding of the epidemiology of violence is needed, including how exposure to physical, sexual, and emotional violence differs by age, sex, and perpetrator. Effective prevention efforts may vary depending on the type of violence, the perpetrator, and the age and sex of those exposed. For example, strategies for preventing physical violence against primary school-aged boys may differ substantially from those for preventing sexual violence against adolescent girls. Similarly, interventions addressing IPV will differ from those addressing violence by parents or guardians. To date, no data synthesis has been conducted in LAC to comprehensively explore the patterns of violence among boys and girls of various ages or the relative perpetrator composition.

Drawing on data from a previously published, large-scale, global data synthesis effort (14), this paper: (i) summarizes available age-, sex-, and perpetrator-specific data on violence in childhood in LAC; (ii) presents age- and sex-specific estimates of the prevalence of physical, sexual, and emotional violence by perpetrator groups; and (iii) discusses gaps in age-, sex- and perpetrator-specific data, comparability, and implications for effective monitoring. Given these objectives and the complexity of violence in LAC, this analysis did not aim to capture all forms of violence against children. For example, organized crime is an important driver of violence in LAC, but it is difficult to quantify (15) and has not been adequately measured due to methodological challenges. Furthermore, inclusion in this review was limited to prevalence estimates that were age-, sex-, and perpetrator-specific. Hence, this paper does not represent all available data on violence and children in LAC. While this paper adds to the epidemiologic understanding of violence in childhood, findings should complement qualitative data that describe wider contexts and types of violence.

## MATERIALS AND METHODS

A systematic review and analysis of published literature and large international datasets meeting eligibility criteria were performed. The protocol is registered in PROSPERO 2015:CRD42015024315, and global findings have been published (14).

### Search strategy

MEDLINE® (United States National Library of Medicine, Bethesda, Maryland, United States), EMBASE (Excerpta Medica Database, Elsevier, Amsterdam, the Netherlands), PsycINFO (American Psychological Association, Washington, DC, United States), Global Health (EBSCO Industries Inc., Birmingham, Alabama, United States), and Web of Science (Clarivate Analytics, Philadelphia, Pennsylvania, United States) were searched for published literature from first record to 7 December 2015, with no language restrictions. Search terms were tailored by database using controlled vocabularies (e.g., MeSH terms for MEDLINE) and included words for violence, children, and study type (observational studies/trials with survey data), described elsewhere (14).

All relevant international datasets known to the authorship team were also included: the Demographic and Health Surveys (16), Reproductive Health Surveys (17), WHO Multi-Country Study on Women’s Health and Domestic Violence (18), Multiple Indicator Cluster Surveys (MICS) (19), Violence against Children Surveys (20), Global School Health Survey (GSHS) (21), Progress in International Reading Literacy Study (22), and Trends in International Mathematics and Science Study (23). Publicly available datasets were also accessed, and if necessary, study representatives requested permission to include them in the analyses.

### Inclusion criteria

Surveys reporting the prevalence of physical, sexual, and emotional violence against children aged 0 – 19 years with age- and perpetrator-specific data from LAC were considered eligible using a priori inclusion criteria. Although the CRC defines children as 0 – 17 years of age, data on 18- and 19-year-olds were included given the ambiguities in age ranges in some datasets (e.g., labeled “≤18 years” or “<19 years”). All definitions of violence and perpetrators were accepted. Only surveys with samples representative of children living in a specific geographic area or school-based populations were considered. Both self-reports and caregiver proxy reports (e.g., from MICS) of violence were included. Data from surveys reporting age bands up to 14 years or recall periods up to 14 years were eligible, but only reports over a narrow age range (5 years or less) were included given the goal of summarizing the prevalence of recent violence. Most estimates were specific to children at each single year of age or had a recall period of 1 year, but those with recall periods less than 1 year (e.g., past-month) were also included.

### Screening and data extraction

Screening of abstracts and full text journal articles was performed by KM and AW. Data on survey characteristics and quality were extracted by KM and LM into a customized Google (Google Inc., Mountain View, California, United States) form database. Each definition of violence, varying considerably across surveys, was recorded.

For the datasets, estimates for age- and sex-specific prevalence of different forms of violence and perpetrators of violence (where applicable) were provided or produced, accounting for the complex sampling scheme employed in each survey.

### Quality appraisal

The quality of estimates was described using a standardized set of criteria developed for this review, including: (a) whether or not a survey was nationally representative, since prevalence can differ within a country’s geographic areas; (b) participation rates and levels of missing data; (c) whether a survey inquired about abstract concepts such as “violence/abuse” rather than behaviorally-specific acts, since the latter avoids the participant’s subjective view of what constitutes violence; (d) whether single or multiple items assessed exposure to violence, since inquiring on multiple, specific acts yields more accurate prevalence estimates; (e) whether an anonymous disclosure method or a face-to-face interview was used, because anonymous methods facilitate disclosure (24); (f) whether a self-report or proxy report was used, since children’s own reports may be more accurate as they age, especially for more hidden or stigmatizing forms of violence—though for very young children, a proxy may be more reliable (25); (g) levels of interviewer training, since greater training results in higher levels of disclosure in surveys on violence against women; and (h) whether violence/maltreatment was the primary focus of the survey or was just a sub-topic of a broader focus, since surveys specific to violence sometimes produce higher disclosure rates.

### Data synthesis

Random effects meta-regressions were performed using Stata®/MP14 (StataCorp LP, College Station, Texas, United States) to estimate the sex-specific prevalence of violence victimization for each year of age (see Annex for sample regression models). Estimates were adjusted for quality-related covariates, including the definition of violence, such that overall estimates would reflect higher-quality surveys with the strongest definitions. The mean estimate and 95% Confidence Interval (95%CI) for each age was plotted separately for children of each sex. Where the prevalence was not reported as a percentage or proportion with a standard error or 95%CI, it was calculated from data in reports or through author communication. Estimates from groups of fewer than 10 participants were excluded.

To produce prevalence estimates by category of perpetrator, estimates with similar perpetrator definitions were combined. For violence by caregivers, MICS questions that measure physical punishment and psychological aggression by caregivers were included. GSHS questions on being physically attacked were included as violence by students, in accordance with how they have been interpreted in previous GSHS data analyses, despite ambiguity in question wording (26, 27). For IPV, a range of definitions (e.g., husband, cohabiting partner, non-cohabiting romantic partner) were included (28). Estimates were adjusted for differences in definition. The population (i.e., the denominator) differed for each category of perpetrator summarized. Estimates of violence by caregivers included the entire population of children; estimates of school violence were computed for children and adolescents in school; estimates of IPV were for ever-partnered adolescents. Thus, the prevalence of each form of violence cannot be directly compared.

For each survey, the child’s age and the recall period for the measure of violence were relevant. Where surveys reported violence over an age range larger than 1 year and up to 5 years, the midpoint of the age range was used and the prevalence was assumed to pertain to that age (e.g., for a sample of 15 – 17-year-olds reporting an average prevalence, the prevalence was assumed to represent students aged 16 years). Estimates with a recall period of less than 1 year were conservatively counted as a past-year prevalence.

### Ethics

All the data analyzed came from the public domain or secondary sources. No ethical clearances were required. Data were anonymized prior to receipt by the study team.

## RESULTS

**FIGURE 1:**
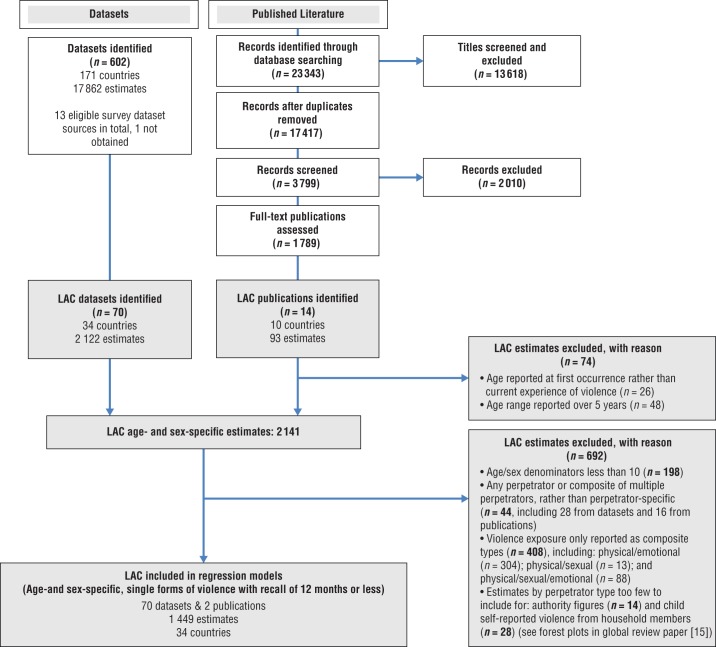
Flowchart for the systematic search of datasets and published literature presenting age-, sex-, and perpetrator-specific estimates of violence against children 0 – 19 years of age in Latin America and the Caribbean (LAC) from first record to December 2015

***Note:*** Boxes in white describe flow diagram for global systematic review of violence against children (15); boxes in grey are specific to this analysis for LAC. “Datasets” refers to a search of all relevant international datasets known to the authorship team. “Published literature” refers to the systematic review of MEDLINE, EMBASE, PsycINFO, Global Health, and Web of Science. “Estimates” describe measures of prevalence of a type of violence specified by age, sex, and perpetrator.

***Source:*** Prepared by the authors from the study results.

The global search yielded 602 datasets and 23 343 publications, of which 70 datasets and 14 publications were specific to LAC (Figure 1). After removing estimates that did not meet inclusion criteria (i.e., prevalence of a type of violence, specified by age, sex, and perpetrator), 1 449 estimates from 72 surveys (70 datasets and 2 publications) across 34 countries in LAC were used in regression models. The Annex lists all countries with available data, and the publications and datasets included.

### Availability of age-, sex- and perpetrator-specific data

Physical violence was the most common form of violence measured by age, sex, and perpetrator (*n* = 827 estimates), followed by emotional violence (*n* = 521 estimates) (Table 1). Far fewer eligible estimates were available for sexual violence (*n* = 101). There were no eligible data on violence by authority figures (e.g., teachers) or gangs/organized crime.

**FIGURE 2:**
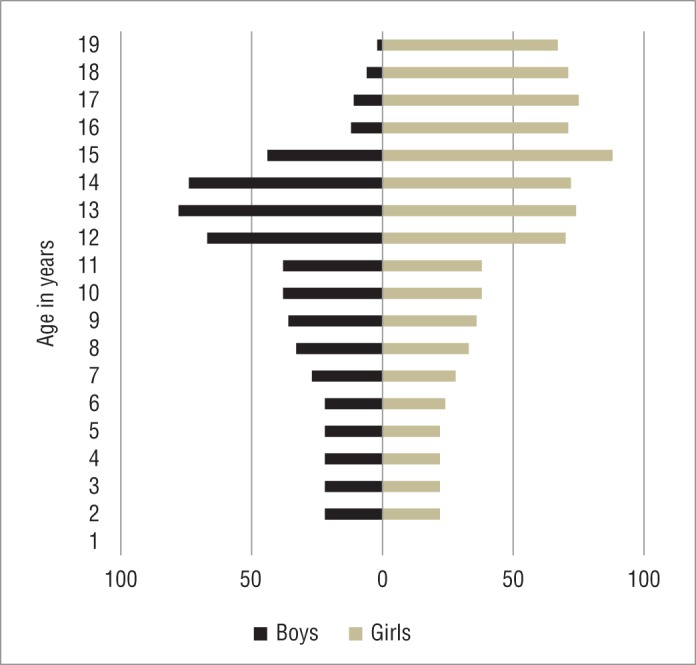
Distribution of prevalence estimates for violence against children 0 – 19 years of age in Latin America and the Caribbean, by age and sex (*n =* 1 449 estimates), from first record to December 2015

***Note:*** “Estimates” describe measures of prevalence of a type of violence specified by age, sex, and perpetrator.

***Source:*** Prepared by the authors from the study results.

Fewer age- and sex-specific prevalence estimates were available for boys (775 estimates from 59 surveys from 31 countries) than girls (1 168 estimates from 72 surveys from 34 countries)—mainly due to the relatively large amount of data on physical and sexual violence against 15 – 19-year-old girls by intimate partners (Figure 2). Few age- and perpetrator-specific estimates were available for boys and girls under 9 years of age or for older adolescent boys (16 – 19 years of age). Confidence Intervals were wide for some prevalence estimates (e.g., violence by other students) due to sparse data. Regarding perpetrator types, no surveys with data on violence by caregivers against children less than 2 years old or over 14 years met inclusion criteria. Data for violence by students meeting inclusion criteria were found only for school-going girls and boys from 8 – 18 years of age. No surveys providing data on IPV and girls less than 15 years of age or boys of any age met inclusion criteria.

### Prevalence of violence by caregivers

**FIGURE 3:**
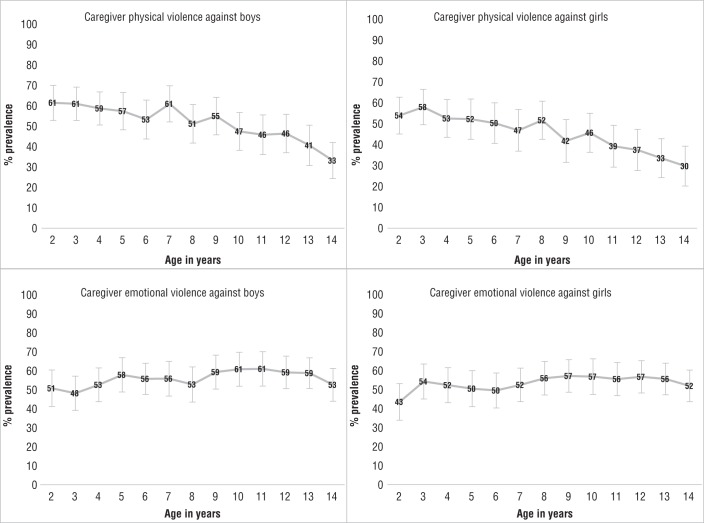
Adjusted prevalence estimates with 95% confidence intervals (bars) for caregiver violence against children in Latin America and the Caribbean: physical and emotional violent discipline by caregivers in the home used against boys and girls, by age

***Source:*** Prepared by the authors from the study results, based on data from the Multiple Indicator Cluster Surveys (19) and Demographic and Health Surveys with MICS questions (16). These are proxy reports by caregivers.

Patterns of violence by caregivers against children across all ages were similar for boys and girls (Figure 3). Physical violence was most commonly reported against children at very young ages, including 50% – 60% of boys and girls aged 2 and 3 years. Physical violence by caregivers declined as age increased, reaching 30 – 40% by age 14 for both girls and boys. Emotional violence followed a somewhat different pattern, with prevalence at 40% – 55% at 2 – 3 years of age for both boys and girls and remaining relatively constant as age increased.

### Prevalence of violence by other students

**FIGURE 4:**
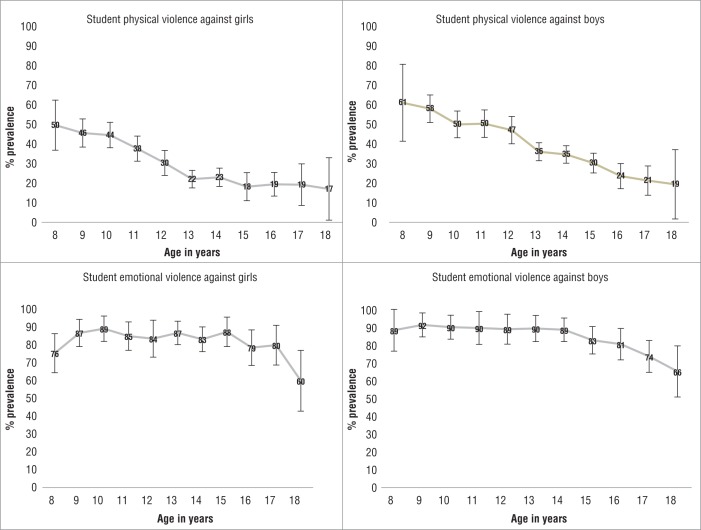
Adjusted prevalence estimates with 95% confidence intervals (bars) for student violence against children in Latin America and the Caribbean: past year student physical and emotional violence against boys and girls, by age

***Note:*** In-school estimates are presented adjusted by violence definitions; physical violence reference definition: “hit or hurt by another student at school.” Emotional reference definition: “something stolen from you at school, made fun of or called names at school, left out of games or activities by other students at school, made to do things you didn't want to do by other students, someone spread lies about you at school.”

***Source:*** Prepared by the authors from the study results, based on data from Global School-based Student Health Surveys (21), Progress in the Evaluation of Educational Achievement (22), Trends in International Mathematics and Science Study (23), and for emotional violence, Serra-Negra, 2015 (29).

At age 8, nearly 50% of girls and 60% of boys had experienced past-year physical violence by another student (Figure 4). Prevalence declined over time, to 17% for girls and 19% for boys by age 18. At 8 years of age, 75% – 90% of boys and girls had experienced past-year emotional violence by other students. Prevalence remained relatively constant over age, reported by 80% – 90% of both boys and girls aged 9 – 17 years. At age 18, data suggest a slight drop in prevalence, although there were fewer data points at this age, resulting in wide Confidence Intervals.

### Prevalence of violence by intimate partners

**FIGURE 5:**
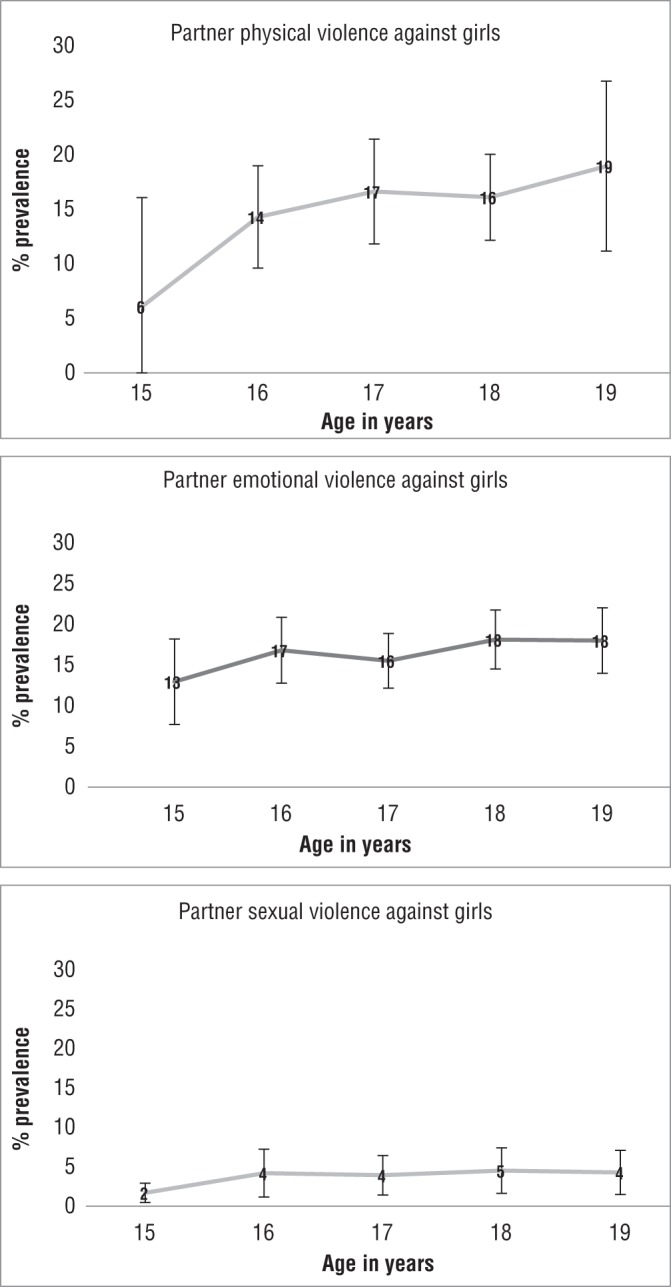
Adjusted prevalence estimates with 95% confidence intervals (bars) for intimate partner violence against children in Latin America and the Caribbean: intimate partner physical, emotional, and sexual violence against ever-partnered girls, by age

***Source:*** Prepared by the authors from the study results based on data from Demographic and Health Surveys (16), Reproductive Health Surveys (17), WHO Multi-Country Study on Women’s Health and Domestic Violence (18), and for physical violence, Blitchtein-Winicki (30).

Among ever-partnered girls 15 – 19 years of age, past-year physical and emotional IPV was reported by 8% – 13% of the 15-year-olds and 15% – 20% of the 16 – 19-year-olds (Figure 5). Sexual IPV was reported by just under 2% of girls 15 years of age and 4% – 5% of girls aged 16 –19 years.

**TABLE 1:** Violence against children in Latin America and the Caribbean: summary of available data that met inclusion criteria, from first record to December 2015

Available data	Number of estimates	Number of countries	Number of surveys
Total	1 449	34	72
Sex			
Male	576	31	56
Female	873	34	70
Form of violence			
Emotional	521	23	35
Physical	827	34	69
Sexual	101	12	13
Main perpetrator groupings			
Household member^a^	572	11	11
Student^b^	299	28	45
Intimate partner	578	12	14
Teacher and authority figure^c^	0	0	0
Gang/organized crime member	0	0	0
Meta regressions			
Physical violence from students, boys	220	27	44
Physical violence from students, girls	219	27	44
Physical violence from household members (proxy reports), boys	143	11	11
Physical violence from household members (proxy reports), girls	143	11	11
Physical violence from intimate partners, girls	102	12	23
Emotional violence from students, boys	70	6	12
Emotional violence from students, girls	69	6	12
Emotional violence from household members (proxy reports), boys	143	11	11
Emotional violence from household members (proxy reports), girls	143	11	11
Emotional violence from intimate partners, girls	96	11	21
Sexual violence from intimate partners, girls	101	12	22

***Note:*** “Estimates” are measures of prevalence of a type of violence specified by age, sex, and perpetrator. “Surveys” are datasets/published literature meeting inclusion criteria.

a All proxy reports included; only one survey included child self-reports, but estimates were too few to be included.

b Global School-based Student Health Survey (21). Student survey data on physical attack and physical fights included in estimates of violence from students.

c No data identified for intimate partner violence against boys.

d One survey met inclusion criteria, but estimates were too few to be included.

***Source:*** Prepared by the authors from the study results.

## DISCUSSION

Findings show that despite the availability of survey data from 34 countries, very few age-, sex-, and perpetrator-specific estimates for some forms of violence exist, including: violence against boys and girls under 9 years of age and older adolescent boys (ages 16 – 19 years); sexual violence against boys of any age and girls under age 15; IPV except for girls ages 15 – 19 years; and violence by specific perpetrator groups, including teachers, other authority figures, and organized crime groups/gangs.

The limited cross-national, age-specific data available for older adolescent boys is of particular concern. Older adolescent boys may be vulnerable to violence by other boys and men related to organized crime or gang involvement (31, 32), as well as non-gang activity (33). The lack of age-specific estimates available in large surveys means that violence against older adolescent boys may receive less attention than it should in prevention and response efforts. Additionally, gaps in data on IPV for girls are notable since child marriage is a concern (34) and is correlated with higher risk of IPV (35); thus, understanding prevalence, co-occurrence, and risk factors for both outcomes would aid policy and programming (36). Gaps in data on sexual violence and by teachers/authority figures will change with expansion of the LAC Violence Against Children Surveys (20). While most LAC countries prohibit physical corporal punishment (and sexual violence) by teachers, prevalence of such violence must be monitored, as some surveys show it to be widespread (14).

Within surveys that met inclusion criteria, past-year physical and emotional violence by caregivers and by other students were common exposures across ages in childhood—both among boys and girls. Physical violence by caregivers and students alike appeared to decline slightly with age, while emotional violence remained fairly constant. IPV against girls aged 15 – 19 years was also substantial, with 15% – 20% of ever-partnered girls reporting past-year physical violence, 15% – 20% reporting emotional violence, and roughly 4% reporting sexual violence.

### Implications

#### Sustainable Development Goals.

Addressing violence against children in LAC requires monitoring progress towards the SDGs. Goal 4 (Quality Education) focuses on ensuring access to quality primary and secondary education and contains a provision for nonviolent educational environments. Under Goal 5 (Gender Equality), Target 5.2 aims to eliminate all violence against women and girls. Under Goal 16 (Peace and Justice), Target 16.2 pledges to end abuse, exploitation, trafficking, and all forms of violence against and torture of children. The 2030 Agenda for Sustainable Development pledges “no one to be left behind.” Discussions have focused on ensuring that all groups make progress towards the SDGs; regarding violence against children, this means that both girls and boys of all ages are considered, as are groups that might be at higher risk of violence. Initiatives, such as those being undertaken by the INSPIRE partners, including WHO, PAHO, United Nations Children’s Fund (UNICEF), the United States Agency for International Development, the United States President’s Emergency Plan for AIDS Relief, the United States Centers for Disease Control and Prevention, Together for Girls, United Nations Office on Drugs and Crime, the Global Partnership to End Violence Against Children, and the World Bank, are critical for drawing attention to violence against girls and boys (36).

#### Prevention programs.

The widespread nature of violence requires multiple approaches. This analysis highlights the need for prevention in both school and home settings, and urgently calls for more data to inform prevention efforts in other settings, such as the community. Prevention in school environments must be targeted as a matter of urgency. The United Nations Educational, Scientific, and Cultural Organization (UNESCO) (37) and the INSPIRE group (38) have issued guidance on effective programming to reduce violence against children, including in schools. Recommended programs have been tested for efficacy on peer violence and bullying (39). The campaign “Safe to Learn” (40)—conceived by the Global Partnership to End Violence Against Children, UNICEF, UNESCO, United Nations Girls’ Education Initiative, and the United Kingdom Department for International Development—will bring renewed attention to ensuring that schools are safe spaces. Several LAC countries are poised to be key partners of Safe to Learn. Evidence suggests that some school-based strategies show promise in prevention of IPV against adolescent girls (41). Further investment is needed for programs aimed at reducing violence in home and community settings.

#### Future research.

Equitable progress reducing violence against girls and boys cannot be effectively monitored with the data available. Existing data on violence by caregivers and students are of limited quality and comparability, highlighting the need for standardization of measures. Ethical and referral protocol for conducting such research must be strengthened, including innovation around safe disclosure mechanisms that allow children to participate in research without experiencing further harm.

Further research and attention are needed to provide reliable estimates of the prevalence and scope of violence stemming from gangs and organized crime. The datasets and sources consulted for this analysis are unlikely to have captured this type of violence, but it is the daily reality of children and their communities in LAC (31, 32). LAC accounts for almost 50% of adolescent homicides despite comprising less than 10% of the global adolescent population; and LAC is the only area in the world where homicide rates among adolescents have increased since 2007 (42). Gang activity is one manifestation of such violence, spurred in part by displacement, high levels of inequality, poverty, urbanization, and drug-trafficking (1, 2). Recent Violence against Children Surveys in El Salvador and Honduras have begun to examine this form of violence, but findings were not available at the time of this analysis. Attention must also focus on how organized crime and community violence affect students traveling to and from school, contribute to school dropout and absenteeism (43), influence those out-of-school, and restrict work access and opportunities for adolescents.

### Limitations

Much available data on violence in LAC did not meet inclusion criteria because surveys measured violence across broad categories of age, for both sexes combined, for composite measures of multiple forms of violence (including poly-victimization), or for any perpetrator. Also, this analysis did not include some key forms of violence, e.g., homicide, trafficking, and organized crime/gangs.

There may have been residual confounding related to violence definitions and survey quality variables, which could partly explain age- and sex-differences in prevalence estimates. Since adjusting for violence definitions and survey quality variables is unlikely to have captured all biases across cultural settings (e.g., unobservable factors), the estimates presented are likely to be lower bounds—i.e., where fewer acts of violence are reported than occur. Under-reporting of certain forms of violence, particularly sexual violence, is likely, due to the stigma associated with victimization and fear of potential reprisals.

Estimates were based on community, household, and school-based surveys, and thus were unlikely to have included children who live outside of family care, out-of-school, on the street, or in institutions. For some forms of violence, data from a limited number of countries were available, which may change once more countries conduct prevalence surveys. Although the literature search did not place restrictions on language, Spanish or Portuguese language journals not indexed by the databases may have been missed. Review of grey literature and reference lists was beyond the scope of the literature search. Finally, there were limited cross-national data examining children’s experiences of violence according to other key vulnerabilities, such as disability, race, or belonging to an indigenous community—hence, these differences were not highlighted in the analyses but are likely to be important (44).

### Conclusions

Data that met inclusion criteria were identified from 34 countries in LAC, and mostly measured physical violence, followed by emotional violence. These data demonstrate the widespread nature of physical and emotional violence by caregivers, by students at school, and by intimate partners of older adolescent girls.

Recommendations are to expand high-quality data collection efforts to monitor progress toward achieving the SDGs; to inform prevention efforts in school, home, and community settings; and to provide insight into forms of violence not yet adequately captured, such as crime- and gang-related activity.

#### Author contributions.

KD designed the study, conducted data analysis, drafted the manuscript, and obtained funding. KM conducted the systematic review and data analysis, and supported the drafting of the manuscript. LK gave input to into the study design, conducted data analysis, performed the overall data synthesis, and drafted the graphics and annexes. SB, AG, BBR, and CH obtained funding and supported the drafting of the manuscript. MP provided input into the statistical methods and data synthesis, and conducted data analysis. LM conducted the systematic review and data analysis. AW assisted with the systematic review. AP, CC, and SK contributed and/or analyzed data. NA gave input to the study design. All authors had input into the writing of the manuscript and approved the final version. KD had full access to all of the study data and had final responsibility for the decision to submit for publication.

#### Acknowledgements.

We gratefully acknowledge all the children and adolescents who participated in the original surveys used in this regional systematic review.

#### Funding.

This work was funded by the Know Violence in Childhood Initiative (CW) and PAHO (KD). MP received funding from the WHO Special Program of Research and Research Training on Human Reproduction. SK received funding from USAID (DHS-7 contract). KM received funding from the National Institute of Mental Health of the National Institutes of Health (award number: F31MH116821-01A1).

No funding sources were involved in gathering of data or analysis for this study, but PAHO staff and consultants (SB, AG, BB, CH) are included as authors. The funders had no role in the study design, data collection or analysis, decision to publish, or preparation of the manuscript.

#### Disclaimer.

Authors hold sole responsibility for the views expressed in the manuscript, which may not necessarily reflect the opinion or policy of the *RPSP/PAJPH*/PAHO and/or the United States National Institutes of Health.

## References

[1] 1. Moser C, Van Bronkhorst B. Youth violence in Latin America and the Caribbean: Costs, causes, and interventions. Washington, DC: World Bank; 1999.

[2] 2. Peetz P. Youth violence in Central America: Discourses and policies. Youth Soc. 2011;43(4):1459-98.

[3] 3. Hillis S, Mercy JA, Amobi A, Kress H. Global prevalence of past-year violence against children: A systematic review and minimum estimates. Pediatrics. 2016;137(3):e20154079. 10.1542/peds.2015-4079 PMC6496958 26810785

[4] 4. Clark CJ, Spencer RA, Everson-Rose SA, Brady SS, Mason SM, Connett JE, et al. Dating violence, childhood maltreatment, and BMI from adolescence to young adulthood. Pediatrics. 2014;134(4):678-85. 10.1542/peds.2014-1179 PMC4179102 25201793

[5] 5. Dube SR, Anda RF, Felitti VJ, Chapman DP, Williamson DF, Giles WH. Childhood abuse, household dysfunction, and the risk of attempted suicide throughout the life span. Findings from the adverse childhood experiences study. JAMA. 2001;286(24):3089-96. 10.1001/jama.286.24.3089 11754674

[6] 6. Norman RE, Byambaa M, De R, Butchart A, Scott J, Vos T. The long-term health consequences of child physical abuse, emotional abuse, and neglect: A systematic review and meta-analysis. PLoS Medicine. 2012;9(11):e1001349. 10.1371/journal.pmed.1001349 PMC3507962 23209385

[7] 7. Duvvury N, Grown C, Redner J. Costs of intimate partner violence at the household and community levels: An operational framework for developing countries. ICRW; 2004.

[8] 8. Stith S, Rosen K, Middleton K, Busch A, Lundeberg K, Carlton R. The intergenerational transmission of spouse abuse: A meta-analysis. J Marriage Fam. 2000;62(3):640-54.

[9] 9. United Nations. Sustainable Development Goals 2015. Available from: https://sustainabledevelopment.un.org/topics/sustainabledevelopmentgoals Accessed 6 August 2019.

[10] 10. United Nations General Assembly. Convention on the Rights of the Child. Geneva: UN; 1989.

[11] 11. Organization of American States. Childhood and adolescence: Building peaceful environments. Unified Resolution. XXI Pan American Child and Adolescent Congress. Washington, DC: OAS; 2014.

[12] 12. Organization of American States. Declaration on violence against and exploitation of children. Resolution of the 44^th^ General Assembly, Organization of American States. Washington, DC: OAS; 2014.

[13] 13. Global Initiative to End Corporal Punishment of Children. Progress towards prohibiting all corporal punishment in Latin America and the Caribbean. Available from: http://endcorporalpunishment.org/wp-content/uploads/legality-tables/Latin-America-and-Caribbean-progress-table-commitment.pdf Accessed 6 August 2019.

[14] 14. Devries K, Knight L, Petzold M, Merrill KG, Maxwell L, Williams A, et al. Who perpetrates violence against children? A systematic analysis of age and sex specific data. BMJ Pediatrics. 2018;2:e000180. 10.1136/bmjpo-2017-000180 PMC5842994 29637183

[15] 15. Muggah R, Tobon KA. Citizen security in Latin America: Facts and figures. Brazil: Igarape Institute; 2018. Available at: https://igarape.org.br/wp-content/uploads/2018/04/Citizen-Security-in-Latin-America-Facts-and-Figures.pdf Accessed 6 September 2019.

[16] 16. ICF. Available Datasets. The DHS Program. Available from: https://dhsprogram.com/data/available-datasets.cfm Accessed 12 August 2019.

[17] 17. United States Centers for Disease Control and Prevention. Reproductive Health Surveys. U.S. Department of Health & Human Services. Available from: https://www.cdc.gov/reproductivehealth/global/tools/surveys.htm Accessed 12 August 2019.

[18] 18. World Health Organization. WHO multi-country study on women's health and domestic violence against women. Geneva; WHO. Available from: https://www.who.int/reproductivehealth/publications/violence/24159358X/en/ Accessed 12 August 2019.

[19] 19. United Nations Children’s Fund. Multiple Indicator Cluster Surveys (MICS). UNICEF. Available from: https://www.unicef.org/statistics/index_24302.html Accessed 12 August 2019.

[20] 20. United States Centers for Disease Control and Prevention. Violence Against Children Surveys (VACS). U.S. Department of Health & Human Services. Available from: https://www.cdc.gov/violenceprevention/childabuseandneglect/vacs/index.html Accessed 12 August 2019.

[21] 21. World Health Organization. Global School-Based Student Health Surveys (GSHS). WHO. Available from: https://www.who.int/ncds/surveillance/gshs/en/ Accessed 12 August 2019.

[22] 22. National Center for Education Statistics. Progress in International Reading Literacy Study (PIRLS). Available from: https://nces.ed.gov/surveys/pirls/ Accessed 12 August 2019.

[23] 23. National Center for Education Statistics. Trends in International Mathematics and Science Study (TIMSS). Available from: https://nces.ed.gov/timss/ Accessed 12 August 2019.

[24] 24. Devries KM, Mak JYT, Garcia-Moreno C, Petzold M, Child JC, Falder G, et al. The global prevalence of intimate partner violence against women. Science. 2013;340:1527-8. 10.1126/science.1240937 23788730

[25] 25. Hamby S, Finkelhor D. The victimization of children: Recommendations for assessment and instrument development. J Am Acad Child Adol Psych. 2000;39(7):829-40. 10.1097/00004583-200007000-00011 10892224

[26] 26. Granero R, Poni E, Escobar-Poni B, Escobar J. Trends of violence among 7th, 8th and 9th grade students in the state of Lara, Venezuela: The Global School Health Survey 2004 and 2008. Ar Public Health. 2011;69(7). 10.1186/0778-7367-69-7 PMC3436614 22958602

[27] 27. Peyton R, Ranasinghe S, Jacobsen K. Injuries, violence, and bullying among middle school students in Oman. Oman Med J. 2017;32(3):98-105. 10.5001/omj.2017.19 PMC5397083 28439379

[28] 28. Garcia-Moreno CJ, Ellsberg H, Heise M, Watts L. WHO multi-country study on women's health and domestic violence against women. Geneva: WHO; 2005.

[29] 29. Serra-Negra JM, Paiva SM, Bendo CB, Fulgencio LB, Lage CF, Correa-Faria P, et al. Verbal school bullying and life satisfaction among Brazilian adolescents: Profiles of the aggressor and the victim. Compr Psychiatry. 2015;57:132-9. 10.1016/j.comppsych.2014.11.004 25465652

[30] 30. Blitchtein-Winicki D, Reyes-Solari E. Factores asociados a violencia fisica reciente de pareja hacia la mujer en el Peru, 2004-2007. [Factors associated to recent intimate partner physical violence against women in Peru, 2004-2007]. Rev Peru Med Exp Salud Publica. 2012;29(1):35-43. 10.1590/s1726-46342012000100006 22510905

[31] 31. Gentle-Genitty C, Kim J, Yi E, Slater D, Reynolds B, Bragg N. Comprehensive assessment of youth violence in five Caribbean countries: Gender and age differences. J Hum Behav Soc Environ. 2017:27(7):745-59.

[32] 32. Reynolds B, Rigby K, Brathwaithe N. The risk factors to youth violence: findings from the CARICOM youth crime and violence school survey. Proceedings of the Caribbean Public Health Agency 60th Annual Scientific Meeting. Kingston: The University of the West Indies; 2015. Pp. 1-75. Bahamas. 2015.

[33] 33. Cunningham W, McGinnis L, Garcia Verdu R, Tesliuc C, Verner D. Youth at risk in Latin America and the Caribbean: Understanding the causes, realizing the potential. Washington, DC: The International Bank for Reconstruction and Development/The World Bank; 2008.

[34] 34. United Nations Children's Fund. UNICEF Global Databases. New York: UNICEF; 2016.

[35] 35. United Nations Children's Fund. Ending child marriage: Progress and prospects. New York: UNICEF; 2014.

[36] 36. World Health Organization. INSPIRE: Seven strategies for ending violence against children. Geneva: WHO; 2016.

[37] 37. United Nations Educational, Scientific and Cultural Organization. School violence and bullying: Global status and trends, drivers and consequences. Paris: UNESCO; 2018.

[38] 38. World Health Organization. INSPIRE Handbook: Action for implementing the seven strategies for ending violence against children. Geneva: WHO; 2018. Available from https://apps.who.int/iris/handle/10665/311034 Accessed 12 August 2019.

[39] 39. Atienzo E, Bazter S, Kaltenthaler E. Interventions to prevent youth violence in Latin America: a systematic review. Int J Public Health. 2017;62:15-29. 10.1007/s00038-016-0909-6 PMC5288433 27766375

[40] 40. The Global Partnership to End Violence Against Children. Safe to Learn. Available from: https://www.end-violence.org/safetolearn Accessed 12 August 2019.

[41] 41. Peterman A, Bleck J, Palermo T. Age and intimate partner violence: an analysis of global trends among women experiencing victimization in 30 developing countries. J Adol Health. 2015;57(6):624-30. 10.1016/j.jadohealth.2015.08.008 26592331

[42] 42. United Nations Children's Fund. A familiar face: Violence in the lives of children and adolescents. New York: UNICEF; 2017.

[43] 43. Aldana J. Strong schools and communities initiative: Working together to build safe schools and protective learning environments. UNICEF Latin America and Caribbean Regional Office; 2015. Available from: https://gbc-education.org/wp-content/uploads/2018/09/Safe_Schools_LA.pdf

[44] 44. Office of the Special Representative of the Secretary-General on Violence Against Children. Toward a world free from violence: Global survey on violence against children. New York: UN; 2013. Available from: https://sustainabledevelopment.un.org/content/documents/2461Towards_a_world_free_from_Violence.pdf Accessed 12 August 2019.

